# Supplementary material for: SpCas9- and LbCas12a-Mediated DNA Editing Produce Different Gene Knockout Outcomes in Zebrafish Embryos
Source: Genes (Basel). 2020 Jul 3;11(7):740. doi: 10.3390/genes11070740 (PMC7397077; doi:10.3390/genes11070740)
Supplement: Supplementary file 1 [file genes-11-00740-s001.pdf]

Table S1. Sequences and scores of target sites used in this work.

Group	Name	Sequence	On-Target Score [21]	Off-Target Score [22]	Number of Analyzed Embryos
LbCas12a	slc6a4a 1	TGCTGCTCTCCCCCGTACTC	NA	49.6	9
	slc6a4a 2	AGGAGATGTACTGCGATAAC	NA	49.5	7
	slc6a4a 3	TGATCCTTCTCCGGAACGCT	NA	49.9	5
	slc6a4 b1	GAGGACCCGGGGCACAGAGG	NA	49.7	9
	slc6a4 b2	CTCCATTTATCGCGGGACTC	NA	49.9	5
	slc6a4 b3	TTTTATCGGTCATTGGATTT	NA	48.3	7
SpCas9	slc6a4a 4	ATGATGAATCAAGAGTACGG	78.6	48.5	10
	slc6a4a 5	TGCGGGCACTGGGACGGACA	66.1	48.6	10
	slc6a4a 6	CAGAGTCCTAAATGTTCCAG	75.2	46.6	10
	slc6a4 b4	TTGGAGGACCCGGGGCACAG	67.7	48.5	10
	slc6a4 b5	CGCCGGGTACAACAGCAACC	61.6	49.5	10
	slc6a4 b6	ATTGGATTTGCGGTAGACCT	62.7	48.3	10

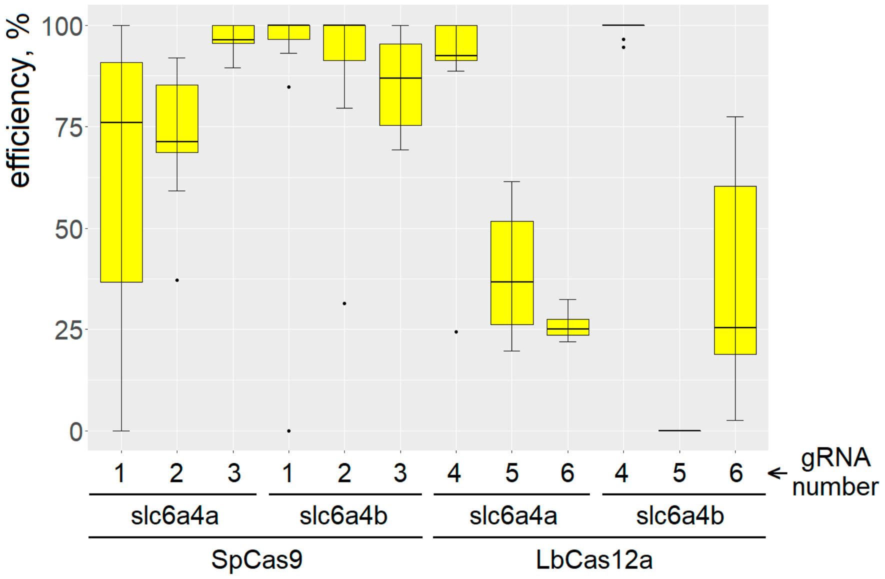

Figure S1. Efficiencies of editing by different groups of RNP complexes. LbCas12a with slc6a4b-crRNA2 did not demonstrate any editing efficiency in any embryo of the group.
